# Supplementary material for: Cilastatin Modulates DPEP1- and IQGAP1-Associated Neuro-Glio-Vascular Inflammation in Oxaliplatin-Induced Peripheral Neurotoxicity
Source: Cells. 2025 Aug 20;14(16):1294. doi: 10.3390/cells14161294 (PMC12385093; doi:10.3390/cells14161294)
Supplement: Supplementary file 1 [file cells-14-01294-s001.zip › cells-3780129-supplementary.pdf]

SUPPLEMENTARY MATERIAL

Table S1. Intensity of specific fluorescence signal for DPEP1, GFAP, CD31, TNFα, IL-6 and IQGAP1 in DRG cell types and subcellular localization (cytosol, nuclei and plasma membrane (p.membr.)) from control, control + cilastatin (CCil) oxaliplatin-treated (OxPt) and OxPt + cilastatin treated (OxPtCil) groups of rats. Intensity levels: - no staining, ± basal-faint, + low, ++ medium, and +++ high.

Cell type		**Neurons**			**Glia**			**Endothelial**		
Localization		Cytosol	Nuclei	Membr.	Cytosol	Nuclei	Membr.	Cytosol	Nuclei	Membr.
Marker	*Condition*									
DPEP1	*Control*	++/+++	+/++	++/+++	+/++	++/+++	++/+++	+/++	+++	+/++
	CCil	++/+++	+/++	++/+++	+/++	+/++	++/+++	+/++	++/+++	+/++
	OxPt	++/+++	++	+++	+/++	++/+++	++/+++	+/++	+++	+/++
	OxPtCil	++/+++	+/++	++/+++	+/++	+/++	++/+++	+/++	+++	++/+++
GFAP	*Control*	-	-	-	+/++	-	+/++	-	-	-
	CCil	-	-	-	++/+++	-	++/+++	-	-	-
	OxPt	-	-	-	+/++	-	+/++	-	-	-
	OxPtCil	-	-	-	++/+++	-	++/+++	-	-	-
CD31	*Control*	-	-	-	-	-	-	+/++	-	+/++
	CCil	-	-	-	-	-	-	+/++	-	+/++
	OxPt	-	-	-	-	-	-	++/+++	-	++/+++
	OxPtCil	-	-	-	-	-	-	++/+++	-	++/+++
TNFα	*Control*	+	-	+	±/+	-	±	++/+++	-	++/+++
	CCil	+	-	+	±/+	-	±	++/+++	-	++/+++
	OxPt	++/+++	±	++/+++	+/++	-	+/++	++	-	++
	OxPtCil	+	-	+	±/+	-	±	++/+++	-	++/+++
IL-6	*Control*	±	-	±	±	-	-	+/++	-	+/++
	CCil	±/+	-	±/+	+/++	-	+/++	+/++	-	+/++
	OxPt	±/+	-	±/+	±/+	-	±	+/++	-	+/++
	OxPtCil	±/+	-	±/+	±/+	-	±	+/++	-	+/++
IQGAP1	*Control*	+/++	-	++/+++	+/++	-	++/+++	+/++	-	+/++
	CCil	+/++	-	+++	+/++	-	+/++	+/++	-	+/++
	OxPt	+	-	+++	++/+++	-	++/+++	+/++	-	+/++
	OxPtCil	+/++	±	++/+++	++/+++	-	++/+++	+/++	-	+/++

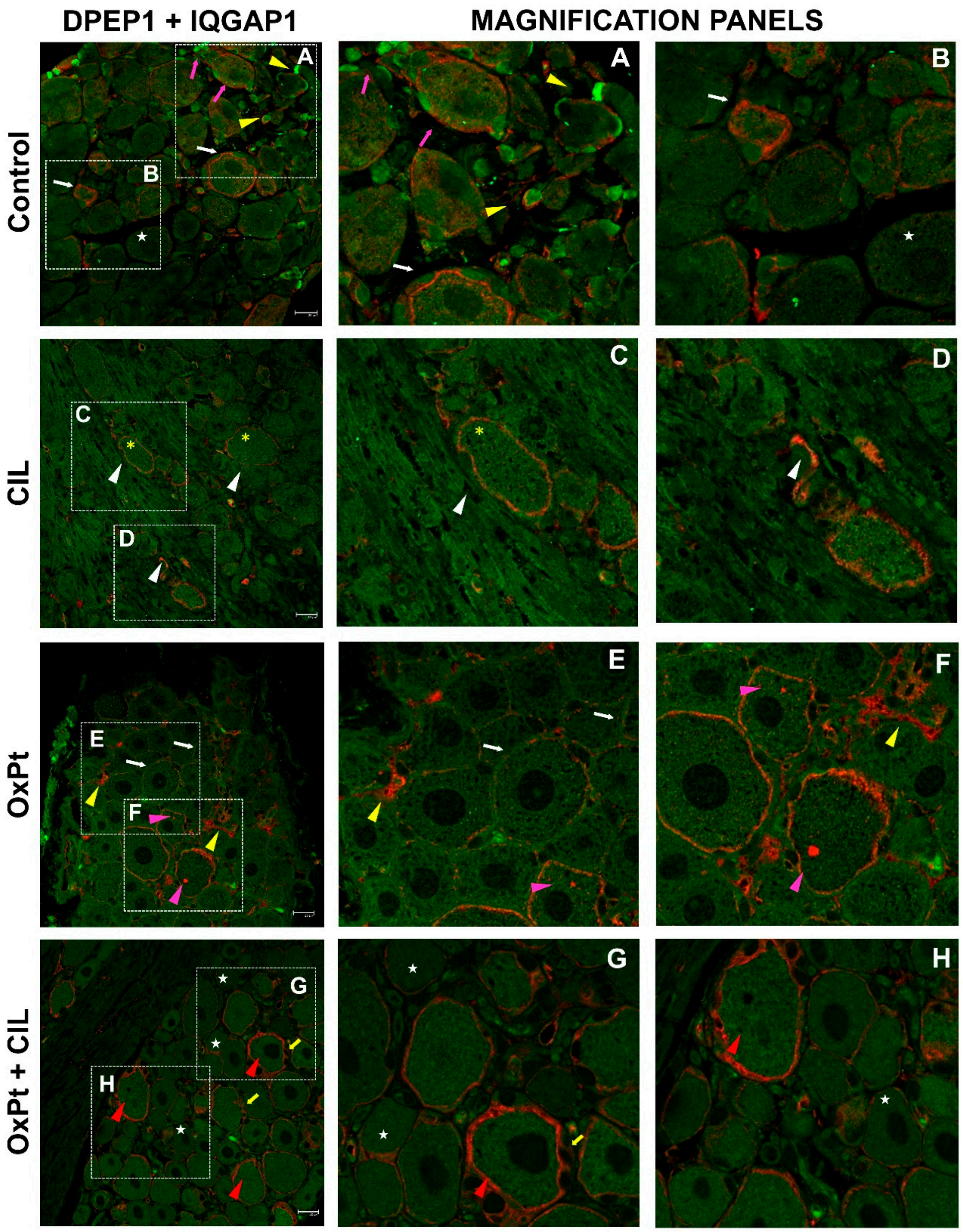

Figure S1. DPEP1 (green) and IQGAP1 (red) magnification panels. Magnified views of selected regions from the main figure (Figure 3) panels presented in the manuscript.

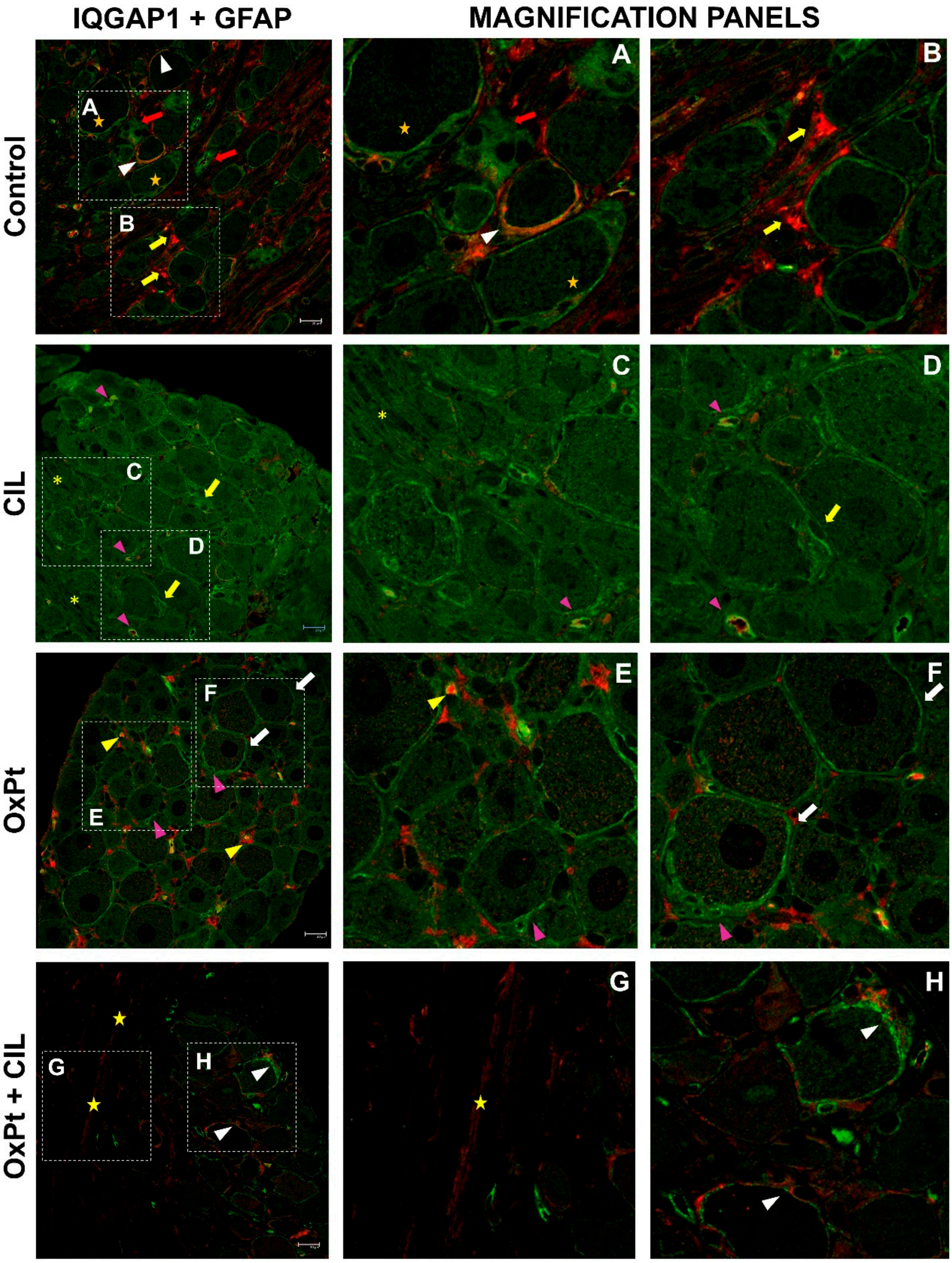

Figure S2. IQGAP1 (green) and GFAP (red) magnification panels. Magnified views of selected regions from the main figure (Figure 4) panels presented in the manuscript.

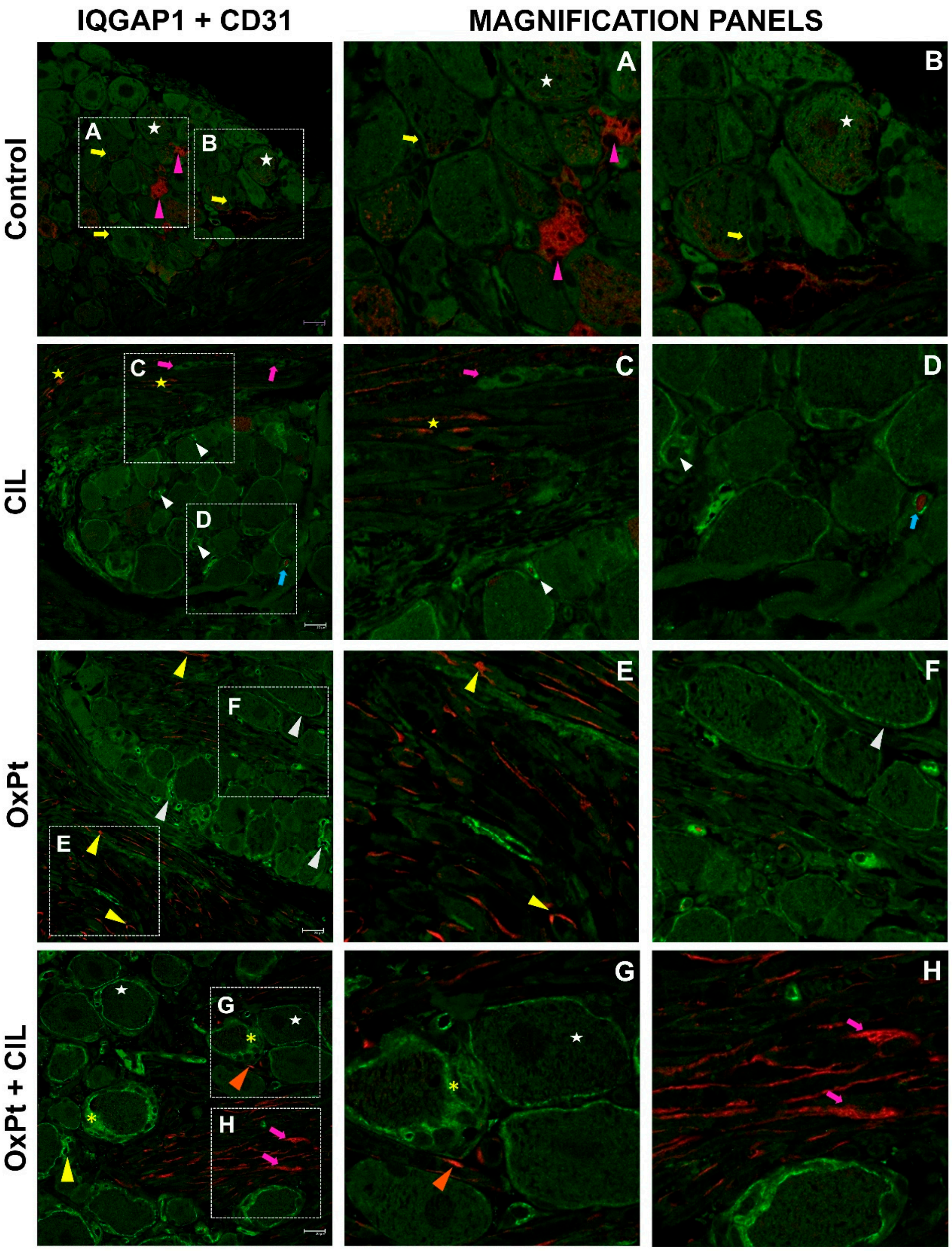

Figure S3. IQGAP1 (green) and CD31 (red) magnification panels. Magnified views of selected regions from the main figure (Figure 5) panels presented in the manuscript.

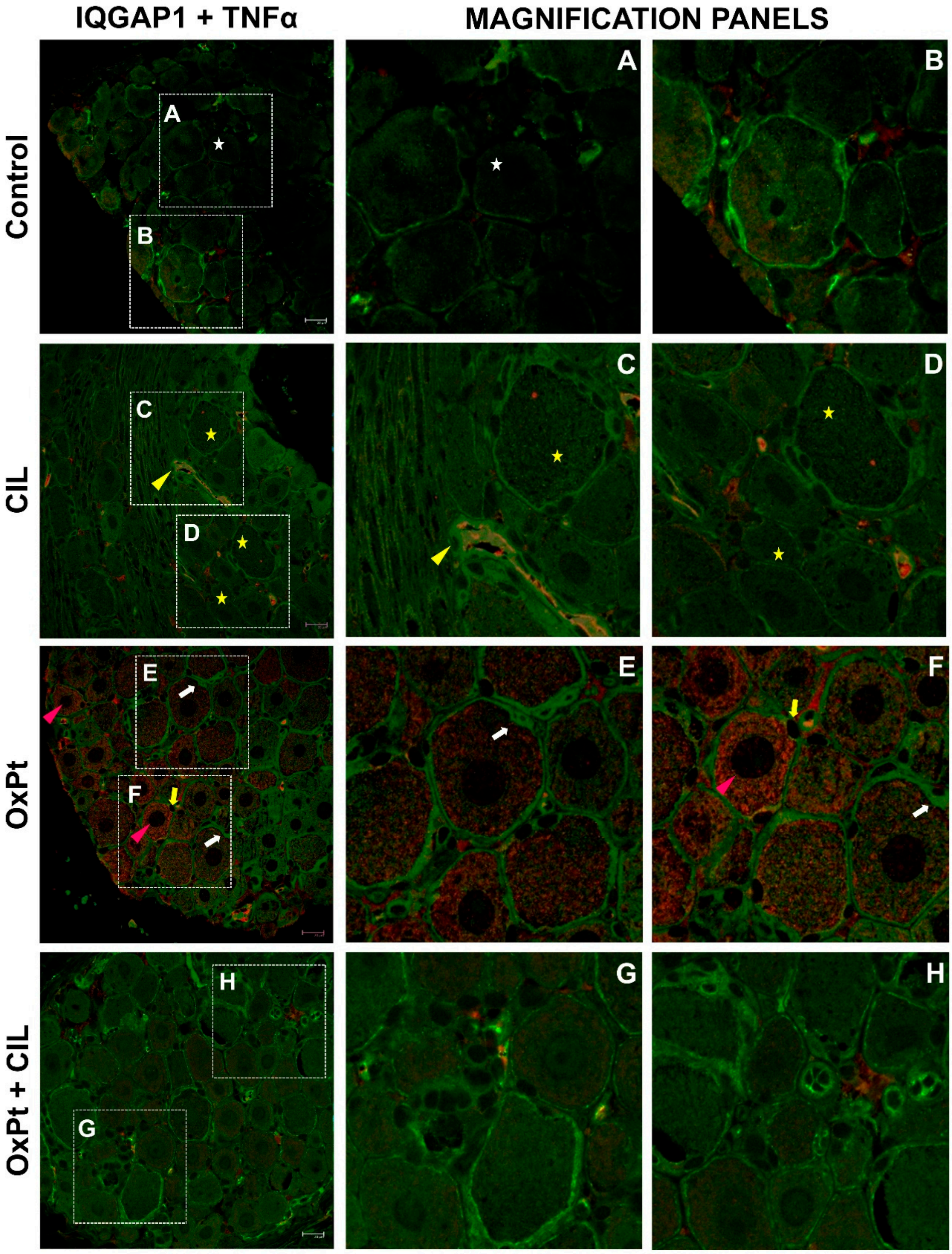

Figure S4. IQGAP1 (green) and TNFα (red) magnification panels. Magnified views of selected regions from the main figure (Figure 6) panels presented in the manuscript.

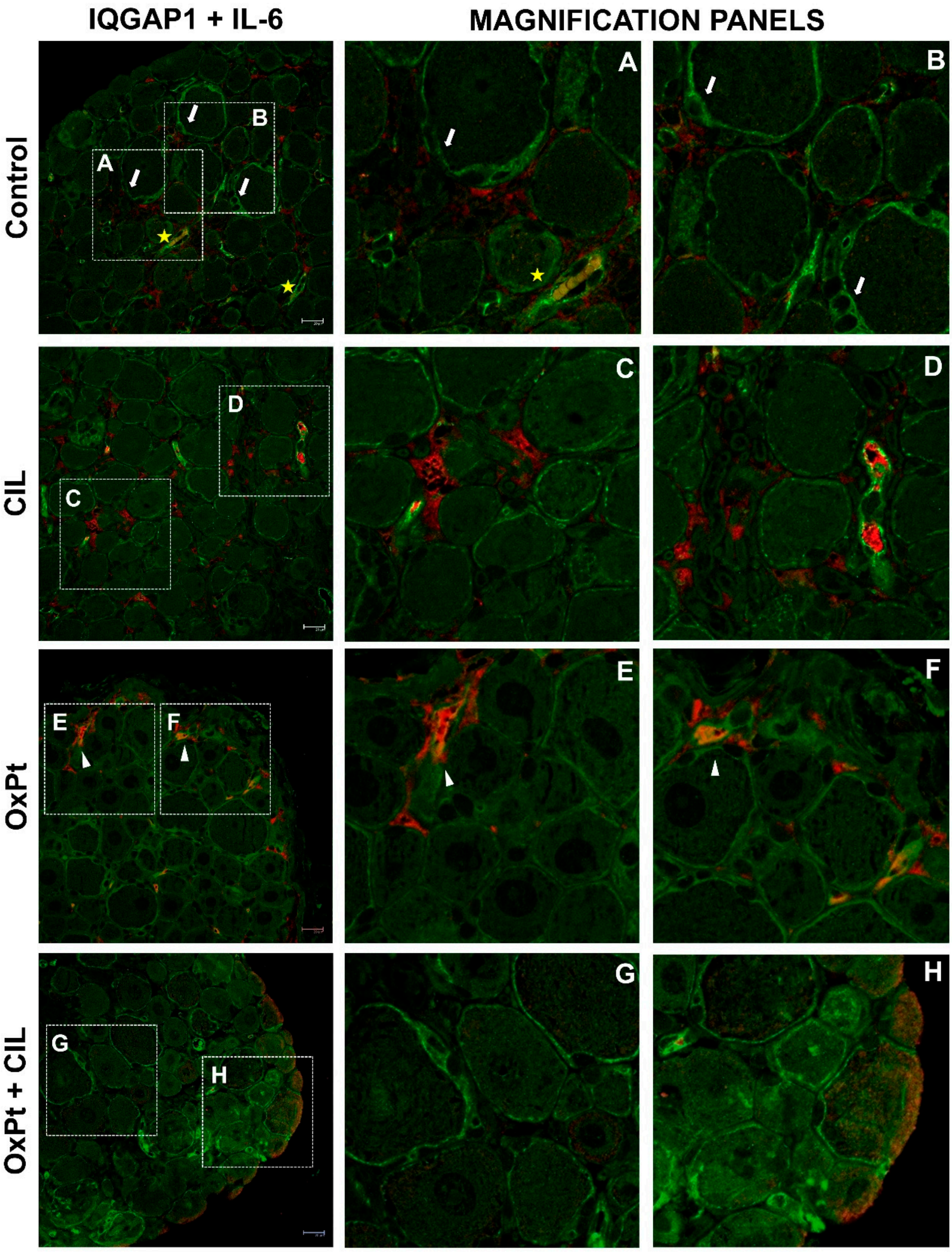

Figure S5. IQGAP1 (green) and IL-6 (red) magnification panels. Magnified views of selected regions from the main figure (Figure 7) panels presented in the manuscript.
